# Variable Syndromic Immunodeficiency in Patients with Biallelic *PRIM1* Mutations

**DOI:** 10.1007/s10875-024-01733-6

**Published:** 2024-05-22

**Authors:** Vasil Toskov, Petra Kaiser-Labusch, Min Ae Lee-Kirsch, PRIM1 study group, Christine Wolf, Carsten Speckmann, Stephan Ehl, Oliver Wegehaupt

**Affiliations:** 1 Clinic of Pediatric Hematology, Oncology and Stem Cell Transplantation, Medical Center, Faculty of Medicine, University of Freiburg, Freiburg, Germany; 2 Prof. Hess Children’s Hospital, Klinikum Bremen-Mitte, Gesundheit Nord gGmbH, Bremen, Germany; 3 Department of Pediatrics, Medizinische Fakultät Carl Gustav Carus, Technische Universität Dresden, Dresden, Germany; 4 Institute for Immunodeficiency, Center for Chronic Immunodeficiency (CCI), Medical Center, Faculty of Medicine, University of Freiburg, Breisacher Str. 115, 79106 Freiburg, Germany

**Keywords:** PRIM1, primordial dwarfism, immunodeficiency, agammaglobulinemia, B cell deficiency, type I interferon, interferonopathy

## Abstract

Mutations in genes of the DNA polymerase complex have been linked to impaired immunological function next to distinct syndromic features. Biallelic mutations in PRIM1 are associated with a primordial dwarfism syndrome with variable hypogammaglobulinemia. The disease is mostly lethal in infancy due to pulmonary infections as well as hepatic cirrhosis. We studied 3 novel patients with PRIM1-deficiency with a focus on immunological consequences. All three shared dysmorphic features including a prominent forehead, triangular face and bilateral cryptorchidism. P1 carried the novel homozygous PRIM1 splice variant c.103+2T>G, allowing residual protein expression and associated with a mild clinical phenotype. P2 and P3 carried the known homozygous variant c.638+36C>G and died in infancy. Paradoxically, B cell lymphopenia was most pronounced in P1. No other significant lymphocyte abnormalities were detected. Interestingly, all 3 patients showed variable, but intermittently excessive Type I interferon signatures. In summary, the B-cell deficiency in PRIM1-deficiency is markedly variable and the severity of syndromic manifestations is not predictive of the immunological phenotype. We highlight a potential contribution of pathological type I interferon activation to disease pathogenesis which warrants further investigations.

**Supplementary Information:**

The online version contains supplementary material available at 10.1007/s10875-024-01733-6.

**To the editor:** Mutations in several genes of the large DNA polymerase complex such as *POLA1, POLD* and *POLE* have been linked to impaired immunological function next to distinct syndromic features. Manifestations of immunodeficiency include lymphopenia with recurrent infections and sterile multiorgan inflammation [1–3]. Since the encoded proteins take part in the DNA replication process, mutations in these essential genes must allow some residual protein function to ensure the cell cycle integrity and survival. In a recent report by *Parry *et al., biallelic mutations in the gene *PRIM1* encoding the catalytic subunit of the DNA primase as part of the DNA polymerase complex in 5 patients were linked to a distinct primordial dwarfism syndrome defined by growth retardation, microcephaly and developmental delay [4]. It also showed characteristics of an immunodeficiency with variable hypogammaglobulinemia. The disease was lethal in infancy mostly due to pulmonary and systemic infections as well as hepatic cirrhosis [4].

Here we add 3 novel patients to show that B-cell deficiency in PRIM1 deficiency is markedly variable and that the severity of the syndromic manifestations is not indicative of the immunological phenotype and its clinical consequences. Three patients with PRIM1 deficiency in our care (Fig. 1A) presented with a clinical course of varying severity. All three shared the characteristic dysmorphic features previously described (prominent forehead, triangular face, hypertelorism, small low-set ears, flat nasal bridge, straight horizontal mouth and bilateral cryptorchidism) [4].

**Fig. 1:**
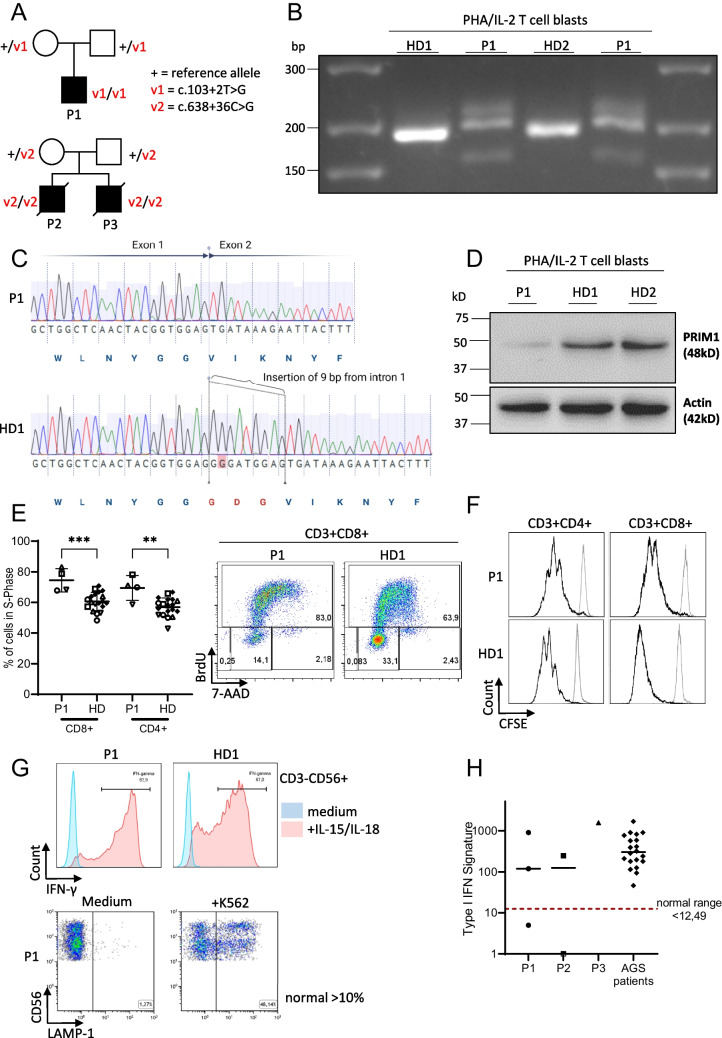
Genetic and immunological features of 2 patients with known and 1 patient with novel homozygous PRIM1 mutations. **A** Pedigrees of two unrelated families with PRIM1 patients. “ + “ denotes the reference PRIM1 allele, V1 and V2 the mutated variants. **B** PRIM1 specific RT-PCR of RNA isolated from P1 and healthy donor (HD) PBMCs that were stimulated with PHA and IL-2 for 3 days. Primers cover the intronic region between exon 1 and exon 2 (WT 187 bp). The two lanes labeled P1 were loaded with the same sample. P1 has a larger transcript due to the insertion shown in (C). Other weaker bands are presumably unspecific transcripts due to strong stimulation with PHA/IL-2. **C** Sanger sequencing reads of cDNA covering the Exon1/2 boundary of PRIM1 from P1 and a HD. The PRIM1 variant c.103 + 2 T > G in P1 causes a 9 bp insertion. Illustration created with BioRender. **D** Immunoblotting of whole cell lysates of P1 and HD PBMCs that were stimulated with PHA and IL-2 for 3 days. **E** BrdU cell cycle assay. Left panel: percentage of CD4 + and CD8 + T-lymphocytes in S-Phase of P1 and HD after 3 days of stimulation with PHA and IL-2. Four independent stimulation experiments with cells from P1 and healthy controls are shown. Individual experiments are depicted by different open symbols. Black symbols represent values from HD obtained in independent experiments. Statistics: ordinary unpaired one-way ANOVA, significance level *p* < 0.05. Right panel: representative FACS plots of cell cycle distribution in CD3 + CD8 + cells. **F** CFSE dilution assay of PBMC incubated for 5 days with medium (gray lines) or PHA (black lines) gated on CD4 + or CD8 + T cells. **G** Upper part: IFN-γ production of CD3-CD56 + NK cells in response to stimulation with IL-15/IL-18 in comparison to a healthy control. Lower part: CD107 expression of NK cells upon stimulation of PBMC with NK-sensitive target K562 cells. **H** IFN signatures in PBMCs of patients P1, P2 and P3. Shown is the fold increase in the IFN score compared with historical positive controls (patients with Aicardi-Goutières Syndrom (AGS)). An IFN score of 12.49 (red dashed line) indicates the median of 10 healthy controls plus 2 SD

P1 is the 2.5 year-old son of non-consanguineous parents who inherited a novel homozygous intronic *PRIM1* variant (c.103 + 2 T > G). No other mutations in genes causing immunodeficiency were detected by whole exome sequencing. He presented at the age of 8 months with bilateral pneumonia and agammaglobulinemia. Since the initiation of immunoglobulin replacement therapy (IRT), no further severe infections have occurred. Despite the dysmorphic features, cranial magnetic resonance imaging showed no abnormalities and at 17 months of age, P1 has so far attained his developmental milestones. He is relatively macrocephalic with head circumference above the 50th percentile and short in stature with his length 2.6 standard deviations below the 1st percentile. There is no evidence of lung or liver disease including a normal pulmonary CT scan. P2 and P3 were siblings with syndromic manifestations including microcephaly as in the cases published by *Parry *et al*.*, as well as severe multisystem organ pathology [4]. P2 suffered from recurrent lower-airway infections with patchy bilateral consolidation of the lungs, bilateral basal ganglia calcification and diffuse hypoechogenic liver islets. Both died due to pneumonia with respiratory failure, P2 at the age of nine months, P3 shortly after birth. Genetic testing revealed the known homozygous *PRIM1* variant c.638 + 36C > G.

The novel variant of P1 was situated in a known splice-site, only 1 nucleotide apart from a previously published splice-site mutation. We therefore analyzed the *PRIM1* transcript in P1 cells by RT-PCR (Fig. 1B). We observed a longer transcript compared to controls and Sanger sequencing confirmed an in-frame insertion of three amino acids (Fig. 1C). We hypothesized that the missense variant c.103 + 2 T > G is hypomorphic, as demonstrated for all other described mutations. Indeed, immunoblotting showed a markedly reduced, but residual expression of the PRIM1 protein at baseline (not shown) and after IL-2/PHA stimulation of T cell blasts (Fig. 1D). Upregulation of *PRIM1* mRNA in P1 T cell blasts was marginal in response to IL-2/PHA stimulation (Suppl. Figure 1). To examine the functional relevance of the variant, we performed a BrdU cell cycle analysis in patient T cells. Interestingly, BrdU incorporation upon PHA/IL-2 stimulation of P1 CD4 + and CD8 + T cells was significantly higher compared to healthy controls with a higher percentage of cells in S-Phase (Fig. 1E).

We further characterized the immunodeficiency in P1 (Suppl. Table 2). Among the few reported cases, P1 has the most severe B cell lymphopenia (2–6 cells/µl), which persisted over time. BTK expression in monocytes was normal. The patient had normal T cell counts, percentage of CD45RA^+^ naïve CD4 T cells and T cell receptor (TCR) Vβ repertoire, as well as upregulation of the activation markers CD25 and CD69 upon stimulation with anti-CD3 ± CD28/PHA. We observed normal proliferation results for CD4 + T cells upon stimulation with PHA (Fig. 1F). In contrast to defects in other components of the DNA polymerase complex [2, 5], NK cell numbers, phenotype of NK subpopulations (% CD62L + among CD3-CD56 + and CD3-CD56bright, as well as % CD16 + CD57 + among CD3-CD56 + NK cells), NK cell function as assessed by IFN-γ production in response to stimulation with IL-15/IL-18 and degranulation as measured via CD107 upregulation were not diminished (Fig. 1G). Thus, despite the widespread expression of PRIM1 in immune cells, the isolated B cell lymphopenia was the only feature of a lymphocyte immunodeficiency that we could detect in P1.

In the report by *Parry *et al. and in P2 and P3, the lethal early-onset lung and liver disease remained insufficiently explained by the observed hypo-/ agammaglobulinemia, which was treated by IRT in some patients. Since both organs can be a target of interferon-driven hyperinflammation, we hypothesized that similar to *POLA1*-deficiency, DNA replication stress could lead to the accumulation of endogenous DNA metabolites arising from DNA repair processes at stalled replication forks, which may stimulate cellular nucleic acid sensors to stimulate an excessive interferon response [1]. We found larger percentages of apoptotic cells (BrdU negative, 7AAD low) in P1 compared to healthy controls (not shown), which could correlate with the accumulation of stalled replication forks. Peripheral blood type I interferon signature in P1, P2 and P3 was increased in several, but not all measurements and reached values in the range of Aicardi-Goutières syndrome patients in the absence of obvious infections and further triggers, which we speculate may be disease-relevant. However, there was no clear pattern of the peripheral blood type I interferon signature that allowed a firm conclusion (Fig. 1H).

In summary, we identify a novel hypomorphic *PRIM1* variant c.103 + 2 T > G associated with the most attenuated phenotype of PRIM1 deficiency described so far with no microcephaly or developmental delay and in overall good health under IRT. The consequences of PRIM1 deficiency for cell cycle progression remain incompletely defined. We observed an increased fraction of CD4 + and CD8 + T cells in S-phase, which could result from an impaired replication process due to a dysfunctional PRIM1 protein. *Parry *et al*.* used fibroblasts for cell cycle studies and observed a decreased percentage of cells in the S-Phase in one of their *PRIM1* patients [4]. Fibroblasts proliferate at a much slower rate than T cells, and may have experienced a cell cycle arrest due to replication stress.

Surprisingly, the B cell immunodeficiency does not align with the other manifestations in this disorder. Our patient with the novel splice site variant showed the most severe B cell deficiency and a mild clinical course. In contrast, P3 inheriting c. 638 + 36C > G as well as 2 published patients with the same variant succumbed early to pulmonary disease, while showing a less severe B cell deficiency. The reason for this difference and the mechanistic basis of the selective B cell defect caused by deficiency of the widely expressed PRIM1 protein remains unclear. A potential contribution of pathological type I interferon activation to disease pathogenesis remains a possibility. This should be addressed in further studies, especially since targeted therapies with JAK inhibitors are available. For optimal care, an interdisciplinary team including immunologists, hepatologists, and social pediatricians is warranted for these complex syndromic patients. PRIM1-deficient patients benefit from IRT and should regularly be monitored for signs of chronic lung disease including bronchiectasis and liver cirrhosis.

### Supplementary Information

Below is the link to the electronic supplementary material.

Supplementary file1 (DOCX 72 KB)

## Data Availability

The datasets generated during the current study are available from the corresponding author on reasonable request.
