# Supplementary material for: Variable Syndromic Immunodeficiency in Patients with Biallelic PRIM1 Mutations
Source: J Clin Immunol. 2024 May 22;44(6):129. doi: 10.1007/s10875-024-01733-6 (PMC11108906; doi:10.1007/s10875-024-01733-6)
Supplement: Supplementary file 1 — Supplementary file1 (DOCX 72 KB) [file 10875_2024_1733_MOESM1_ESM.docx]

**Supplemental Data**

**1. Materials and Methods**

*Research subjects*

All families gave their informed consent for this study (Freiburg HILDA Biobank Broad Consent). Genetic testing in P1, P2 and P3 and their respective parents was performed (P1 - Zentrum für Humangenetik Tübingen, Germany, and P2/P3 – MHH, Hannover, Germany) as Trio Whole Exome Sequencing. Healthy donor samples were obtained from the Institute for Transfusion Medicine of the Medical Center Freiburg, Germany.

*Cells and cell culture*

Peripheral blood mononuclear cells (PBMC) were isolated from P3 blood samples. Cells were either cultured in Iscove's Modified Dulbecco's Medium (IMDM, Gibco) supplemented with 10% FCS (anprotec) and 1% penicillin/streptomycin (Life Technologies) only, or stimulated with phytohemagglutinin (Remel, Lenexa (US)) 1% and interleukin-2 200 U/ml (R+D). Cells were harvested on day 3 (72h), counted and used for further experiments.

*RNA isolation, amplification and gel electrophoresis*

Total RNA was isolated from T cell blasts (on day 3 after stimulation) using the RNeasy Micro Kit (Qiagen) according to supplier’s protocol. cDNA was obtained by reverse transcription using the qScript™ cDNA SuperMix (VWR International). PRIM1 specific PCR was performed for cDNA amplification using Primers designed to cover the genomic intronic region between exon 1 and exon 2 (wildtype length 187bp). Forward Primer: ggagacgtttgaccccaccg. Reverse Primer: ctggttgttgaaggattggtagcg. The PCR product was loaded on a 4% agarose gel, 80V 30min, 100V 90 min, in TBE-buffer to separate small size fragments. cDNA sequencing was performed commercially by Azenta Life Sciences using the same primer pair.

*Immunoblotting*

Whole cell lysates were obtained from 1x10^6 PBMCs in 50mM Tris/HCl, 150mM NaCl, 5mM MgCl_2_, 0,1% NP40, 1x complete protease inhibitor (Roche), and 1mM DTT. Lysates were loaded on 12% SDS-Polyacrylamid-gels, resolved (10 min 100V; 80 min 120 V) and transferred to nitrocellulose membranes. Membranes were blocked using 5% milkpowder in PBS-T. The following antibodies were used for Immunoblotting: anti-p48 Primase Rat mAb (8G10; 1:1000; Cell Signaling #4725), anti-beta actin mouse mAb (AC-15; 1:4000; Sigma-Aldrich #A1978), HRP goat anti-rat IgG polyclonal ab (1:5000; Biolegend #405405), HRP anti-mouse IgG (1:10.000; GE HealthCare #NA931-1mL). Detection was performed using Amersham ECL Select Peroxide and Luminol Solutions (RPN2235V2 and RPN2235V1) and chemiluminescence imaging by the Fusion FX (Vilber Lourmat).

*Cell cycle distribution*

Cell cycle phase distribution was examined on day three of stimulation of PBMCs using the FITC BrdU Flow Kit (BD Pharmingen). T cell blasts were labeled with 10 μM BrdU for 60 min before staining was carried out according to supplier’s protocol. The following antibodies were used for Flow Cytometry analysis: anti-BrdU FITC (BD Pharmingen), anti-CD3 PE (HIT3a; BD Pharmingen #555340), anti-CD4 APC Cy7 (RPA-T4; BD Pharmingen #557871), anti-CD8 BV510 (RPA-T8; BD Horizon #563256). For all experiments, FACS was carried out using a Gallios or a Navios Flow Cytometer (Beckman Coulter) and FACS data was analysed using FlowJo software (Becton Dickinson & Company, v10.9.0) or Kaluza software (Beckman Coulter, v2.1).

*T-cell proliferation*

P3 PBMCs were isolated and labeled with 0,5µM CFSE (Molecular Probes, C-1157), resuspended in Iscove's Modified Dulbecco's Medium (IMDM, Gibco) and either cultured in medium or stimulated with 2,5 μg/ml phytohemagglutinin (Remel, Lenexa (US)). T-cells were harvested on day 4 and surface staining was carried out using the following antibodies: anti-CD4 PC7 (SFCI 12T4D11; Beckman Coulter #737660) and anti-CD8 APC (RPA-T8; BD Pharmingen #555369).

*NK cytokine expression*

P3 PBMCs were isolated and cultured in either Iscove's Modified Dulbecco's Medium (IMDM, Gibco) or stimulated with 200 ng/ml IL-15 (Miltenyi Biotec) and 200 ng/ml IL-18 for 24 hours at 37°C, 5% CO2. 5 hours before the end of stimulation, GolgiPlug 1:1.000 (BD Biosciences, #555029) was added to inhibit protein transport. Surface staining was performed using the following antibodies: anti-CD3 PerCP (SK7, BD Pharmingen #345766), and anti-CD56 APC (NCAM16.2, BD Pharmingen #341027). Subsequently the cells were permeabilised, fixed and intracellular staining was performed with an anti-IFN-gamma FITC antibody (B27, BD Biosciences, #554700).

*NK degranulation*

P1 PBMCs were isolated and cultured in either Iscove's Modified Dulbecco's Medium (IMDM, Gibco) only or stimulated with K562 cells for 2 hours at 37°C, 5% CO2. Surface staining was subsequently carried out using the following antibodies: anti-CD107a (LAMP1) PE (H4A3, BD Pharmingen #555801), CD3 PerCP (SK7, BD Pharmingen #345766), and anti-CD56 APC (NCAM16.2, BD Pharmingen #341027).

*Expression of PRIM1 mRNA*

Expression levels of PRIM1 mRNA were examined on day three after stimulation of PBMCs (see cells and cell culture). RNA isolation and cDNA generation was carried out as described above. FS Universal SYBR Green (Sigma-Aldrich Chemie GmbH) was used for detection of DNA synthesis, quantitative PCR was carried by a StepOne Plus Real-Time PCR Systems Cycler (Life Technologies) and analysis was performed by StepOnePlus Software (Life TechnologieTs).

*Real-time quantitative (rtq) PCR of IFN-stimulated genes*

RTq-PCR of IFN-stimulated genes and IFN score analysis was performed as previously described (see Wan, Rensheng et al. “Phenotypic spectrum in recessive STING-associated vasculopathy with onset in infancy: Four novel cases and analysis of previously reported cases.” Frontiers in immunology vol. 13 1029423. 6 Oct. 2022, doi:10.3389/fimmu.2022.1029423).

**2. Supplemental Tables**

**Supplemental Table 1: Clinical findings**

| **ID** | **Gender** | **Genetics** | **Family history** | **Birth and development** | **Respiratory** | **GI** | **Hepatic** | **Endocrine** | **IRT** | **Clinical course** |
| --- | --- | --- | --- | --- | --- | --- | --- | --- | --- | --- |
| P1 | male | homozygous c.103+2T>G | not consanguinous | 38 weeks | bilateral pneumonia | no | no | no | yes | alive, 2 years of age |
| P2 | male | not performed | not consanguinous | 36 weeks, growth retardation | ARDS, chronic cough, home oxygen, RSV bronchiolitis | no | inhomogenous liver | no | yes | death at 9 months, respiratory failure |
| P3 | male | homozygous c.638+36C>G | not consanguinous | 38 weeks, growth retardation, developmental delay | neonatal RDS | n/a | n/a | n/a | n/a | death during neonatal period |
| 1 | female | homozygous c.638+36C>G | consanguinous | 30 weeks, growth retardation, no motor milestones | chronic cough, ARDS, bronchiolitis | no | transaminitis | no | yes | death at 7 months, bronchiolitis |
| 2 | female | homozygous c.638+36C>G | n/a | 38 weeks, growth retardation, developmental delay | no | rotavirus, C. difficile enteritis | no | no | no | alive, 7 years of age |
| 3 | male | homozygous c.638+36C>G | not consanguinous | 35 weeks, growth retardation, developmental delay | ARDS (adenovirus) | recurrent diarrhea | transaminitis, cirrhosis | hypothyroidism | yes, once | death at 2 years, ARDS |
| 4 | male | homozygous c.638+36C>G | not consanguinous, broher of 3 | 38 weeks, growth retardation, developmental delay | home oxygen, ILD, pulmonary hypertension | recurrent vomitting and diarrhea, Rotavirus enteritis | transaminitis, hepatomegaly, hepatic fibrosis | hypothyroidism | yes | death at 19 months, cardiorespiratory failure |
| 5 | male | c.103+1G>T and c.901T>C | not consanguinous | 38 weeks, growth retardation, developmental delay | pneumonitis, recurrent infections, ILD | viral gastroenteritis, pancreatic insufficiency | AP elevated, nodular hyperplasia | no | yes | death at 2 years, viral gastroenteritis |
| Abbreviations: P: patient, n/a: no data, AP: alkaline phosphatase, ARDS: acute respiratory distress syndrome, GI: gastrointestinal, ILD: interstitial lung disease, IRT: immunoglobulin replacement therapy, RSV: respiratory syncytial virus  Note: P1-3 represent the patients reported in the main text. ID 1-5 is a summary of the patient information provided in Parry et al. | | | | | | | | | | |

**Supplemental Table 2: Hematological and immunological findings at diagnosis**

| **ID** | **WBC (G/L)** | **ANC (G/L)** | **PLT (G/L)** | **HB (g/L)** | **IgM (g/L)** | **IgG (g/L)** | **IgA (g/L)** | **Lymphocytes (G/L)** | **CD4+ (G/L)** | **CD8+ (G/L)** | **CD19+ (G/L)** | **CD16/56+ (G/L)** | **CFSE** | **Further immunological tests** |
| --- | --- | --- | --- | --- | --- | --- | --- | --- | --- | --- | --- | --- | --- | --- |
| P1 | 8,7 | 2,54 | 256 | 121 | undetectable | undetectable | undetectable | 4,61 | 2,775 | 1,4 | 0,003 | 0,175 | normal | s. main text |
| P2 | 31,04 | 24,49 | 232 | 100 | 0,61 | undetectable | 0,05 | 3,2 | 1,639 | 0,534 | 0,13 | 0,575 | normal | FoxP3 normal, CD25/CD69 slightly decreased |
| P3 | n/a | n/a | n/a | n/a | n/a | n/a | n/a | n/a | n/a | n/a | n/a | n/a | n/a | n/a |
| 1 | 6,2 | 2,8 | 20 | 55 | undetectable | undetectable | undetectable | 1,8 | n/a | n/a | n/a | n/a | n/a | n/a |
| 2 | 10,19 | 7,2 | n/a | n/a | 0,82 | 3,5 | 1,5 | 2,16 | 1,144 | 0,28 | 0,159 | n/a | n/a | n/a |
| 3 | n/a | n/a | 44 | n/a | 1,08 | 5,38 | 0,09 | 1,96 | n/a | n/a | n/a | n/a | n/a | n/a |
| 4 | 12,07 | 8,31 | 20 | 78 | 0,36 | <3 | undetectable | 1,32 | 0,91 | 0,567 | 0,0118 | 0,29 | n/a | oxidative burst normal |
| 5 | n/a | n/a | 406 | n/a | low/undetectable | low/undetectable | low/undetectable | n/a | normal | normal | nearly absent | normal | n/a | n/a |
| Abbreviations: P: patient, n/a: no data, WBC: white blood count, ANC: absolute neutrophil count, PLT: platelets, HB: hemoglobin, Ig: immunoglobulin, CD: cluster of differentiation, CFSE: carboxyfluoroscein succinimidyl ester  Note: P1-3 represent the patients reported in the main text. ID 1-5 is a summary of the patient information provided in Parry et al. | | | | | | | | | | | | | | |

**3. Supplemental Figures**

**Supplemental Figure 1**

Quantitative *PRIM1* expression in unstimulated PBMCs (medium cultured) and PBMCs additionally stimulated for three days with IL-2 and IL-2/PHA, respectively. Three independent stimulation experiments with PBMCs from P1 (in blue) and healthy controls (in purple) were pooled for the analysis. Bars show the mean, error bars show the standard deviation of values. On the left, *PRIM1* expression in PBMCs is shown as relative to b-actin. On the right, *PRIM1* expression is shown normalised to the medium condition of healthy donors.
